# Restricted Feeding Schedules Modulate in a Different Manner the Expression of Clock Genes in Rat Hypothalamic Nuclei

**DOI:** 10.3389/fnins.2016.00567

**Published:** 2016-12-06

**Authors:** Leonardo D. De Araujo, Silvia L. Roa, Ana C. Bueno, Fernanda B. Coeli-Lacchini, Clarissa S. Martins, Ernane T. Uchoa, Jose Antunes-Rodrigues, Lucila L. Elias, Paula C. Elias, Ayrton C. Moreira, Margaret De Castro

**Affiliations:** ^1^Department of Physiology, University of Sao PauloRibeirao Preto, Brazil; ^2^Department of Internal Medicine, University of Sao PauloRibeirao Preto, Brazil; ^3^Department of Pediatrics of the Ribeirao Preto Medical School, University of Sao PauloRibeirao Preto, Brazil

**Keywords:** circadian oscillators, hypothalamic nuclei, food restriction, clock gene expression, corticosterone

## Abstract

Food access restriction is associated to changes in gene expression of the circadian clock system. However, there are only a few studies investigating the effects of non-photic synchronizers, such as food entrainment, on the expression of clock genes in the central oscillators. We hypothesized that different feeding restriction patterns could modulate the expression of clock genes in the suprachiasmatic nucleus (SCN) “master” clock and in extra-SCN oscillators such as the paraventricular (PVN) and arcuate (ARC) hypothalamic nuclei. *Wistar* rats were divided into four groups: Control group (CG; food available *ad libitum*), Restricted night-fed (RF-n; food access during 2 h at night), Restricted day-fed (RF-d; food access during 2 h at daytime), Day-fed (DF; food access during 12 h at daytime). After 21 days, rats were decapitated between ZT2-ZT3 (0800–0900 h); ZT11-ZT12 (1700–1800 h), or ZT17-18 (2300–2400 h). Plasma corticosterone was measured by radioimmunoassay (RIA). The expression of *Clock, Bmal1, Per1, Per2, Per3, Cry1, Cry2, Rev-erb*α, and *Ror*α were assessed in SCN, PVN, and ARC hypothalamic nuclei by RT-PCR and calculated by the 2^[−DeltaDeltaCT(Cyclethreshold)]^(2^−ΔΔCT^) method. Restricted food availability during few h led to decreased body weight in RF-n and RF-d groups compared to controls and DF group. We also observed an anticipatory corticosterone peak before food availability in RF-n and RF-d groups. Furthermore, the pattern of clock gene expression in response to RF-n, RF-d, and DF schedules was affected differently in the SCN, PVN, and ARC hypothalamic nuclei. In conclusion, the master oscillator in SCN as well as the oscillator in PVN and ARC, all brain areas involved in food intake, responds in a tissue-specific manner to feeding restriction.

## Introduction

Physiological rhythms are regulated by neuronal hypothalamic circadian oscillators (Bass and Takahashi, 2010). Circadian oscillators are kept synchronized one with another and with environmental time by the suprachiasmatic nucleus (SCN), known as “master” clock in mammals. The SCN receives direct photic input from the retina, generates a pronounced circadian rhythm and in turn, synchronizes other subsidiary cellular oscillators (Reppert and Weaver, 2001).

In mammals, the mechanism of cell-autonomous circadian clock depends on two core clock genes, *Clock*, and *Bmal1*, which are transcriptional activators of an auto-regulatory negative feedback loop (Lowrey and Takahashi, 2004). These genes form a heterodimer that activates their target genes, *Period* (*Per*), and *Cryptochrome* (*Cry*), which form a repressor complex in the cytoplasm that returns to the nucleus and interacts with CLOCK-BMAL1 to inhibit their own transcription (Takahashi et al., 2008). *Bmal1* and *Clock* genes also increase the mRNA levels of *Rev-erb*α and *Ror*α, which compete for the binding to the retinoic acid-related orphan receptor response elements (ROREs) repressing or activating the expression of *Bmal1* (Green et al., 2008). Post-translational modifications also modulate these auto-regulatory loops (Lamia et al., 2009; Nakahata et al., 2009; Jang et al., 2012).

Glucocorticoid circadian secretion is modulated via CRH and ACTH periodic release under the SCN control (Dallman et al., 1978, 1993; Buijs et al., 2003; Fahrenkrug et al., 2008). Furthermore, the glucocorticoid release is also modulated by the central circadian clock system in a manner independent of the HPA axis, possibly due to altering the sensitivity of the adrenal cortex to ACTH through the autonomic nervous system (Ishida et al., 2005; Kalsbeek et al., 2006; Nader et al., 2010). In addition to the photoperiod, feeding time is a powerful Zeitgeber of the glucocorticoid circadian rhythm. Rats display an anticipatory peak of glucocorticoid, release 1–2 h before the availability of food (Krieger et al., 1977). This phenomenon seems to be dependent on a distinct food-entrainable hypothalamic oscillator (Stephan et al., 1979; Stephan, 2002). Indeed, glucocorticoids are required for meal-induced changes in the expression of hypothalamic neuropeptides (Uchoa et al., 2012). Paraventricular (PVN) and arcuate (ARC) nuclei have been implicated in the control of energy homeostasis and express orexigenic and anorexigenic peptides. PVN receives projections not only from satiety-related neurons from the nucleus of the solitary tract but also from ARC neurons indicating that the hypothalamus plays an important role in the integrative responses that control food intake (Schwartz et al., 2000).

Although SCN generates a circadian rhythm, the daily rhythm of food intake can also regulate other hypothalamic nuclei, such as PVN, ARC, dorsomedial (DMH), and ventromedial (VMH) nuclei. Previous studies tested the susceptibility of non-photic entrainment cues and demonstrated that restricting food availability with maintenance of photoperiod cues, is a strong Zeitgeber that changes only slightly SCN clock gene expression but can alter *Per1, Per2*, and *Bmal1* expression in the PVN, anterior pituitary, and adrenal tissue (Girotti et al., 2009). In addition, timed hypocaloric feeding was also able to synchronize the temporal organization of SCN clockwork (Caldelas et al., 2005). Moreover, restricted feeding schedules entrain oscillations of *Per1, Per2*, and *Bmal1* expression in different phases among various hypothalamic structures (Mieda et al., 2006; Verwey et al., 2007; Minana-Solis et al., 2009). Furthermore, peripheral clocks, particularly the liver, pancreas, kidney, and heart can be modulated by food restriction (Damiola et al., 2000; Le Minh et al., 2001; Stokkan et al., 2001; Green et al., 2008).

The majority of the studies on the effects of non-photic synchronizers, such as food entrainment, on the expression of clock genes in the hypothalamus used restriction feeding schedule with access to food restricted to a few hours daily during the light phase. In nocturnal animals, restricting feeding time to the dark does not seem to significantly alter the phase angle of the cyclic clock gene expression (Damiola et al., 2000). However, when food is offered only during the light phase, the expression of clock-related genes in the liver becomes inverted (Damiola et al., 2000; Le Minh et al., 2001). Moreover, food restriction, i.e., hypocaloric feeding, seems to cause changes not only in the temporal organization of peripheral oscillators, but also in the circadian SCN rhythm in rodents (Challet, 2007). Therefore, in this study, we investigate whether different restricted feeding schedules during the light phase or during the dark phase can alter clock gene expression in the SCN and other hypothalamic nuclei of rats despite the presence of photoperiodic cues.

## Materials and methods

### Animals and experimental design

This study was approved by the Animal Ethics Committee of the Ribeirao Preto Medical School of University of Sao Paulo, Brazil (Protocol n° 077/2011). Adult male *Wistar* rats weighing about 200 g, were housed for 5 days in individual cages with food and water *ad libitum*, under controlled conditions of temperature, humidity and on a 12:12 h light/dark (LD) cycle, with lights on at 0600 h (Zeitgeber time; ZT 0).

All animals were fed with standard chow. After 5 days of acclimation period, rats were divided into four groups with different dietary patterns for 21 days. Control group (CG, total number of 23 rats): food *ad libitum*, available at all the time; Restricted night-fed (RF-n, total number of 25 rats): access to food restricted to ZT12–ZT14 (1800–2000 h); Restricted day-fed (RF-d, total number of 27 rats): access to food restricted to ZT3–ZT5 (0900–1100 h); Day-fed (DF, total number of 21 rats): access to food from ZT0–ZT12 (0600–1800 h). Water was offered *ad libitum* to all groups. Food intake and body weight were determined every day throughout the experiment. On the 21th day, 6–10 rats were decapitated per time point between 0800 and 0900 h (ZT3), between 1700 and 1800 h (ZT11), or between 2300 and 2400 h (ZT17).

To avoid unspecific or stress-related elevations of corticosterone secretion, animals were handled by the same investigator during the experiment and on the last day animals were decapitated within 60 s. Trunk blood was immediately collected for corticosterone determination by RIA, as previously described (Castro et al., 1995). The assay sensitivity was 0.4 μg/dl, and the inter- and intra-assay variations were 4.8% and 6.7%, respectively. Animals of control group decapitated at ZT3, which showed plasma concentrations of corticosterone above 3 ug/dl were excluded because of undesirable stress condition, as previously published by our laboratory (Laguna-Abreu et al., 2005).

Brain was collected and flash-frozen in a dry ice-isopentene bath at −30°C and stored at −80°C until processing. SCN, PVN, and ARC hypothalamic nuclei were microdissected bilaterally by punch technique in a cryostat according to coordinates from −0.92 to −1.52 (600 μm thicknesses), −0.92 to −2.12 (1200 μm), −2.12 to −3.62 (1500 μm) from the bregma (Paxinos, 1997), respectively, using a stainless steel punch needle of 1.0 mm in diameter for the SCN and 1.5 mm for other nuclei. Tissue samples were transferred to a microtube containing RNA later reagent (Ambion, USA) and stored at −80°C until RNA isolation.

### RNA isolation, cDNA synthesis, and amplification by real-time PCR

Total RNA was isolated from each micropunched hypothalamic tissue sample using TRIzol reagent (Life Technologies) according to the manufacturer's protocol. RNA concentrations were quantified by spectrometry (Nanodrop 2000, Thermo Fisher Scientific Inc., Waltham, MA, USA). RNA integrity was verified by measuring the 28/18S ratio, with an acceptable range of 1.6–2.0 and confirmed by 1.2% agarose gel electrophoresis. mRNA was reverse transcribed from 500 ng of total RNA using the High Capacity cDNA Reverse Transcription kit and MultiScribe® enzyme (Life Technologies).

For semi-quantitative Real-Time PCR (qPCR), TaqMan® assays (Life Technologies, Foster City, CA, USA) were used according to the manufacturer's recommendation using cDNA (diluted 1:5) as template. The specific probes and TaqMan® Gene Expression Assays IDs used were *Clock* (Rn00573120_m1), *Bmal1* (Rn00577590_m1), *Per1* (Rn01325256_m1), *Per2* (Rn01427704_m1), *Per3* (Rn00709499_m1), *Cry1* (Rn01503063_m1), *Cry2* (Rn00591457_m1) *Rev-erb*α (Rn01460662_m1), and *Ror*α (Rn01173769_m1) genes. Each PCR reaction was performed in duplicate. Water, instead of cDNA, was used as a negative control. Housekeeping genes, GAPDH (Rn99999916_s1), and ACTB (Rn00667869_m1), mRNA expression were analyzed for each cDNA sample. For each sample, the threshold cycle (Ct) was determined and normalized to the average of the two housekeeping genes (ΔCt = Ct Unknown − Ct Housekeeping genes). The determination of gene transcript levels in each sample was obtained by the 2^−ΔΔCt^ method (Livak and Schmittgen, 2001). The median value obtained to each sample of tissues from animals submitted to different dietary pattern was compared with the median value obtained from control rats decapitated at ZT3 (0900 h).

### Statistical analysis

Quantitative variables were expressed as mean and standard error (X ± SEM). Differences among morning, afternoon, and night values in the same feeding schedule or differences among rats of different groups at the same time were analyzed by non-parametric analysis of variance of Kruskal-Wallis with Dunn's *post-test*. Analyses were performed using the GraphPad Prism 5.0 (GraphPad, San Diego, CA). Statistical significance was considered at *P* < 0.05.

## Results

### Body weight and food intake

**Figure 1:**
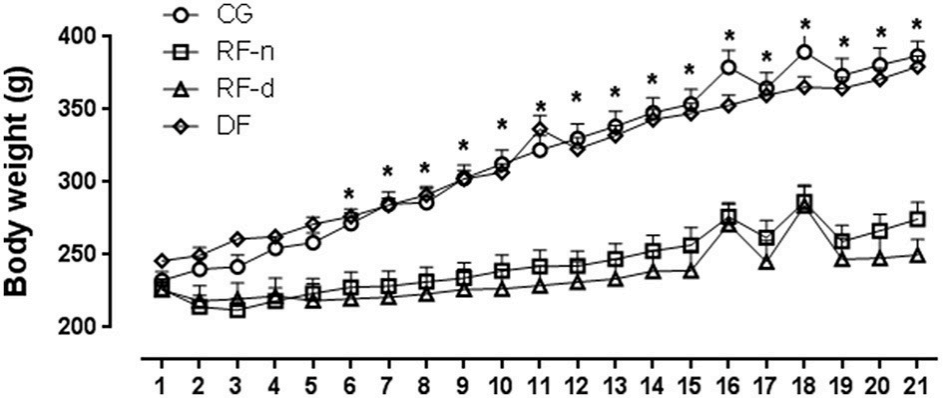
**Evolution of the body weight from Day 0–21 in the control group (circles) with food ***ad libitum***, restricted night-fed group (RF-night, squares) with access to food restricted to ZT12 to ZT14 (1800–2000 h), restricted day-fed group (RF-day, triangles) with access to food restricted to ZT3 to ZT5 (0900–1100 h) and, restricted day-fed group (DF, diamonds) with access to food restricted to ZT0 to ZT12 (0600–1800 h)**. *N* = 6–10 rats per time point per group. ZT, Zeitgeber Time. Significant difference among ZTs within the same feeding condition: ^*^P < 0.05.

All groups presented similar body weight at the beginning of the experiment. After 21 days, decreased body weight (*P* < 0.01) was observed in RF-n (274.5 ± 59.3 g) and RF-d (250.0 ± 56.0 g) groups compared with CG (387.0 ± 49.1 g) and DF groups (379.5 ± 35.7 g); no difference was observed between RF-n and RF-d groups as well as between CG and DF groups (Figure 1). Similarly, daily food intake (g) and consequently lower caloric intake were observed (*p* < 0.0001) in RF-n (15.2 ± 3.0) and RF-d (14.3 ± 2.6) groups compared with CG (30.7 ± 3.8) and DF (25.0 ± 2.8) groups; no difference was observed between RF-n and RF-d groups as well as between CG and DF groups. Of note, even after normalization of food intake to body weight, RF-n and RF-d groups still presented smaller ingestion than CG and DF groups.

### Plasma corticosterone levels

**Figure 2:**
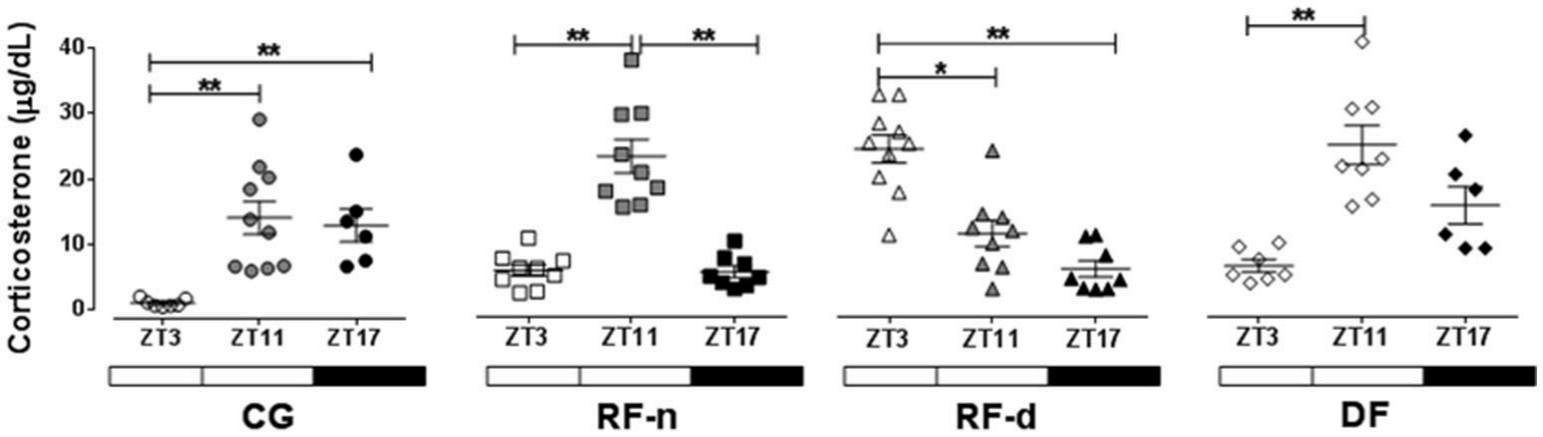
**Plasma corticosterone (μg/dl) in the control group (circles) with food ***ad libitum***, restricted night-fed group (RF-night, squares) with access to food restricted to ZT12 to ZT14 (1800–2000 h), restricted day-fed group (RF-day, triangles) with access to food restricted to ZT3–ZT5 (0900–1100 h) and, restricted day-fed group (DF, diamonds) with access to food restricted to ZT0 to ZT12 (0600–1800 h) at ZT3, ZT11, and, ZT17 (black symbols), corresponding to 900, 1700, and 2300 h; respectively**. *N* = 6–10 rats per time point per group. ZT, Zeitgeber Time. Significant difference among ZTs within the same feeding condition: ^*^P < 0.05, ^**^P < 0.01.

Figure 2 shows the plasma corticosterone levels (μg/dl) in CG, RF-d, RF-n, and DF groups at ZT3, ZT11, and ZT17. CG presented higher corticosterone levels at ZT11 (14.1 ± 8.0; *P* < 0.01) and ZT17 (12.9 ± 6.2; *P* < 0.01) compared with ZT3 (1.0 ± 0.6), with no difference between ZT11 and ZT17. The RF-n group showed higher corticosterone levels at ZT11 (20.7 ± 7.6, *P* < 0.01) compared with ZT3 (3.6 ± 2.6) and ZT17 (3.5 ± 2.6) with no difference between ZT3 and ZT17. On the other hand, RF-d group showed an inverted daily pattern of corticosterone secretion compared with CG and RF-n groups with higher levels at ZT3 (22.7 ± 6.2) compared with ZT11 (10.6 ± 5.7) and ZT17 (5.6 ± 3.3; *P* = 0.0002) with no difference between ZT11 and ZT17. The DF group showed higher corticosterone levels at ZT11 (11.6 ± 4.0, *P* < 0.01) compared with ZT3 (2.9 ± 1.6) and ZT17 (7.3 ± 3.3) with no difference either between ZT3 and ZT17 or between ZT11 and ZT17.

### Gene expression

**Figure 3:**
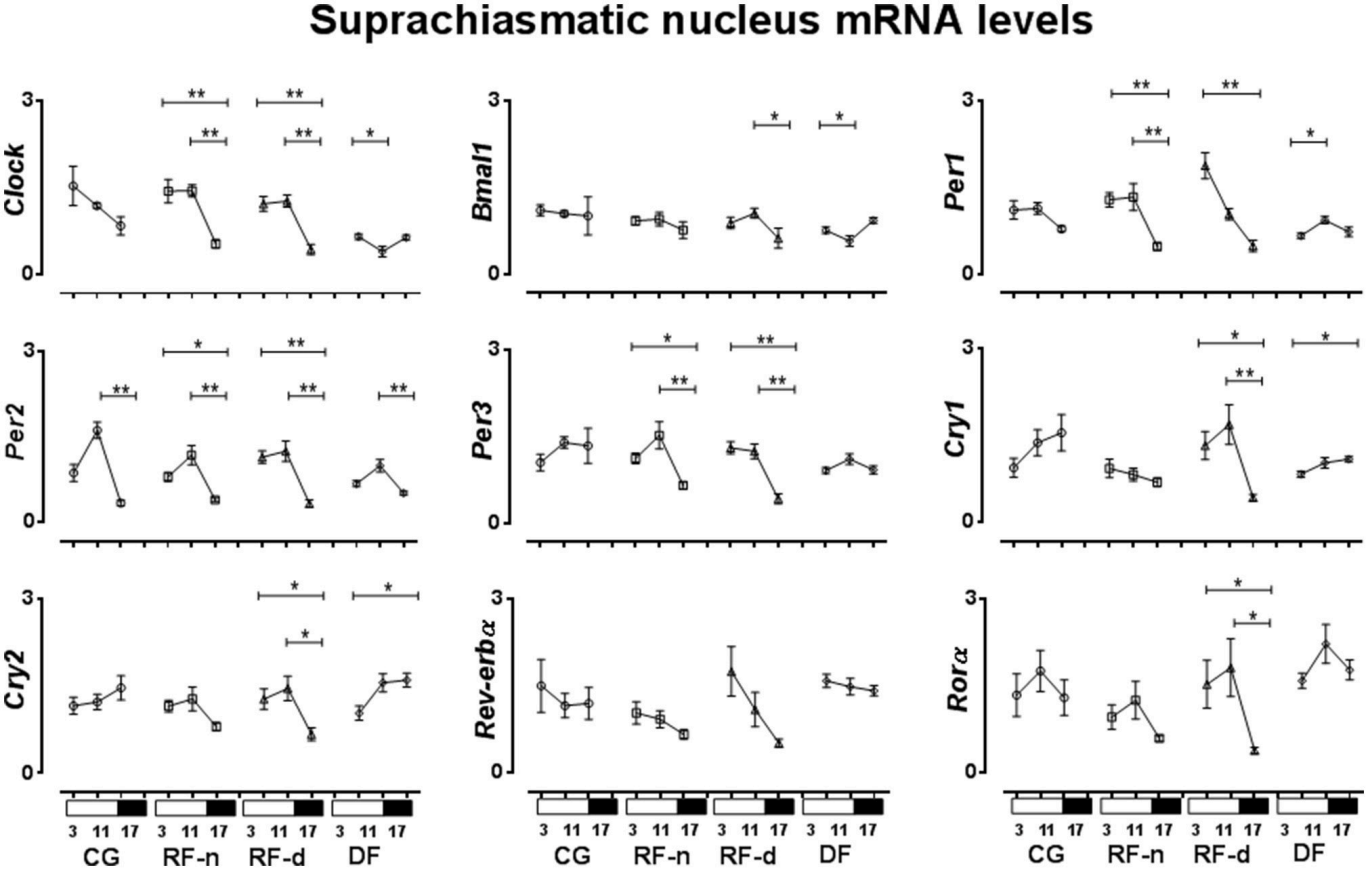
**Relative expression of clock genes in Suphrachiasmatic nucleus (SCN) in the control group, with food ***ad libitum***; restricted night-fed group (RF-night), with access to food restricted to ZT12–ZT14 (1800–2000 h); restricted day-fed group (RF-day), with access to food restricted to ZT3–ZT5 (0900–1100 h); and day-fed group (DF), with access to food restricted to ZT0–ZT12 (0600–1800 h) at ZT3, ZT11, and ZT17, corresponding to 0900, 1700, and 2300 h; respectively**. *N* = 6–10 rats per time point per group. ZT, Zeitgeber Time. Significant difference among ZTs within the same feeding condition: ^*^P < 0.05, ^**^P < 0.01.

The expression of clock genes in the SCN of CG, RF-n, RF-d, and DF groups at ZT3, ZT11, and ZT17 are presented in Figure 3. Differences among morning, afternoon, and night values in the same feeding schedule showed no difference in *Rev-erb*α expression among ZT3, ZT11, and ZT17 in any group. In CG, no differences were observed in the mRNA expression of *Clock, Bmal1, Per1, Per3, Cry1, Cry2*, and *Ror*α among ZT3, ZT11, and ZT17. In DF group, there were no differences in the expression of *Per3* and *Ror*α in any ZT, while higher gene expression were observed for *Clock* at ZT3 compared with ZT11 (*P* = 0.02), for *Bmal1* at ZT17 compared with ZT11 (*P* = 0.01), for *Cry1*, and *Cry2* at ZT17 compared with ZT3 (*P* = 0.03 and *P* = 0.02, respectively), and for *Per1* at ZT11 compared with ZT3 (*P* = 0.02). Regarding *Per2*, the highest expression was observed at ZT11 and the lowest at ZT17 in all groups (*P* < 0.01). The expression of *Clock, Per1*, and *Per3* genes did not show differences at ZT3, ZT11, and ZT17 in CG; however, in RF-d and RF-n groups, these genes presented higher expression in the morning and in the afternoon (*P* < 0.01). Compared with CG and RF-n groups, in which food access occurred without dissociation with the usual pattern of rat nocturnal activity, higher expression of *Bmal1* was observed at ZT11 (*P* = 0.05) in the RF-d group, while *Cry1* (*P* < 0.01), *Cry2* (*P* = 0.01), and *Ror*α at ZT3 and ZT11 (*P* < 0.01).

**Figure 4:**
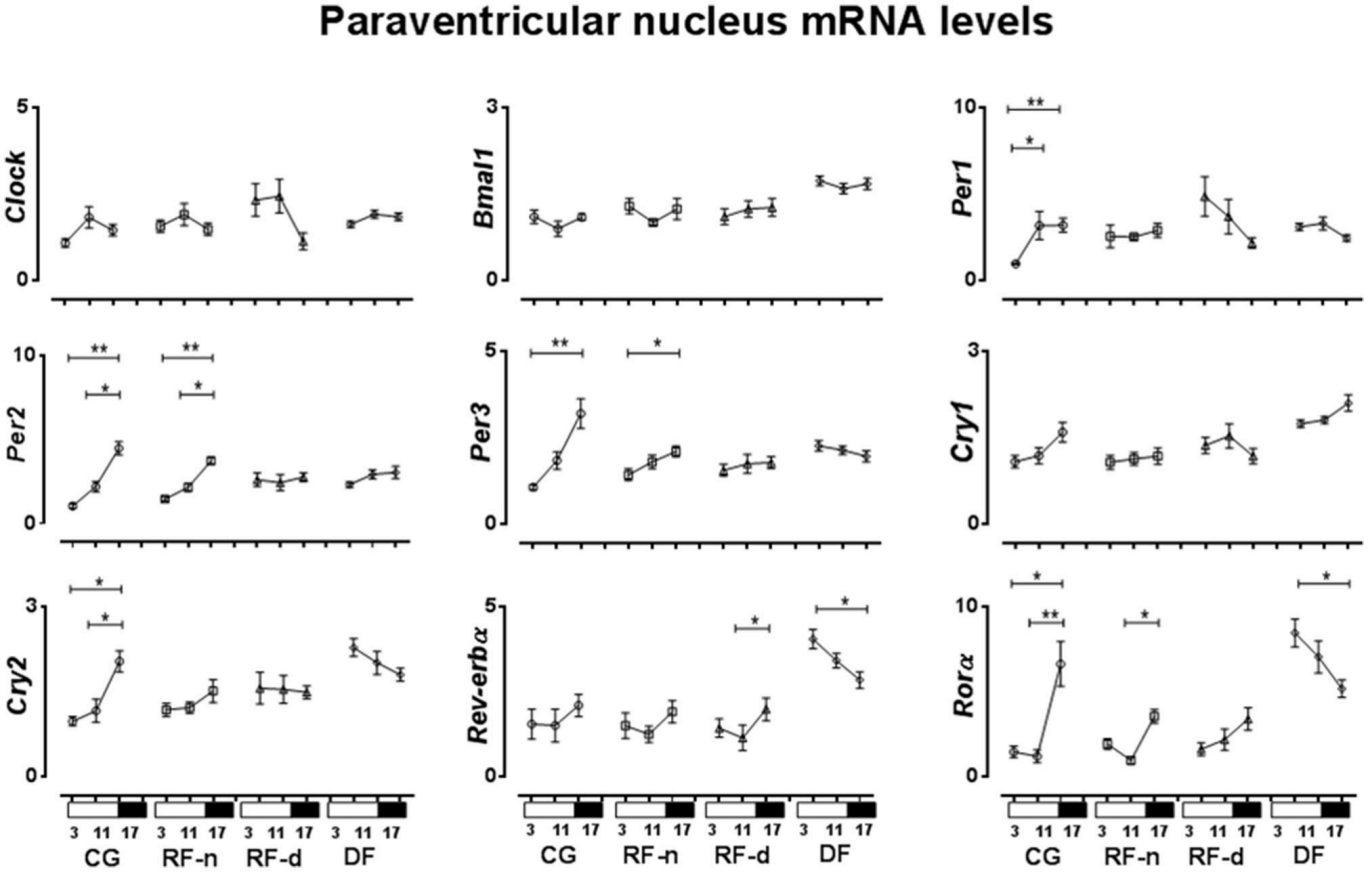
**Relative expression of clock genes in Paraventricular nucleus (PVN) in the control group, with food ***ad libitum***; restricted night-fed group (RF-night), with access to food restricted to ZT12 to ZT14 (1800–2000 h); restricted day-fed group (RF-day), with access to food restricted to ZT3 to ZT5 (0900–1100 h); and day-fed group (DF), with access to food restricted to ZT0 to ZT12 (0600–1800 h) at ZT3, ZT11, and ZT17, corresponding to 0900, 1700, and 2300 h; respectively**. *N* = 6–10 rats per time point per group. ZT, Zeitgeber Time. Significant difference among ZTs within the same feeding condition: ^*^P < 0.05, ^**^P < 0.01.

The expression of clock genes in the PVN of CG, RF-n, RF-d, and DF groups at ZT3, ZT11, and ZT17 are presented in Figure 4. Regarding differences among morning, afternoon, and night values in the same feeding schedule, we observed no differences in the expression of *Clock, Bmal1*, and *Cry1* genes at ZT3, ZT11, and ZT17 in any studied group. In the CG, higher *Per1* expression was observed in the afternoon (ZT11) and at night (ZT17) (*P* < 0.01), while higher expression of *Per2, Per3*, and *Ror*α (*P* < 0.01) were observed at night (ZT17). No different *Rev-erb*α expression was observed among the three different ZT points. In the RF-n group, the pattern of expression of *Per2* (*P* < 0.01), *Per3* (*P* = 0.04), and *Ror*α (*P* < 0.01) genes was similar to CG, with higher expression at night (ZT17), while *Per1* and *Cry2* diurnal variation observed in the CG was abolished in this group. In the RF-d group, there was no difference in the expression of the studied genes among ZT3, ZT11, and ZT17, with exception of *Rev-erb*α, which had higher expression at night (ZT17) (*P* = 0.03). DF group showed higher expression of *Ror*α and *Rev-erb*α at ZT3 compared with ZT17 (*P* = 0.02).

**Figure 5:**
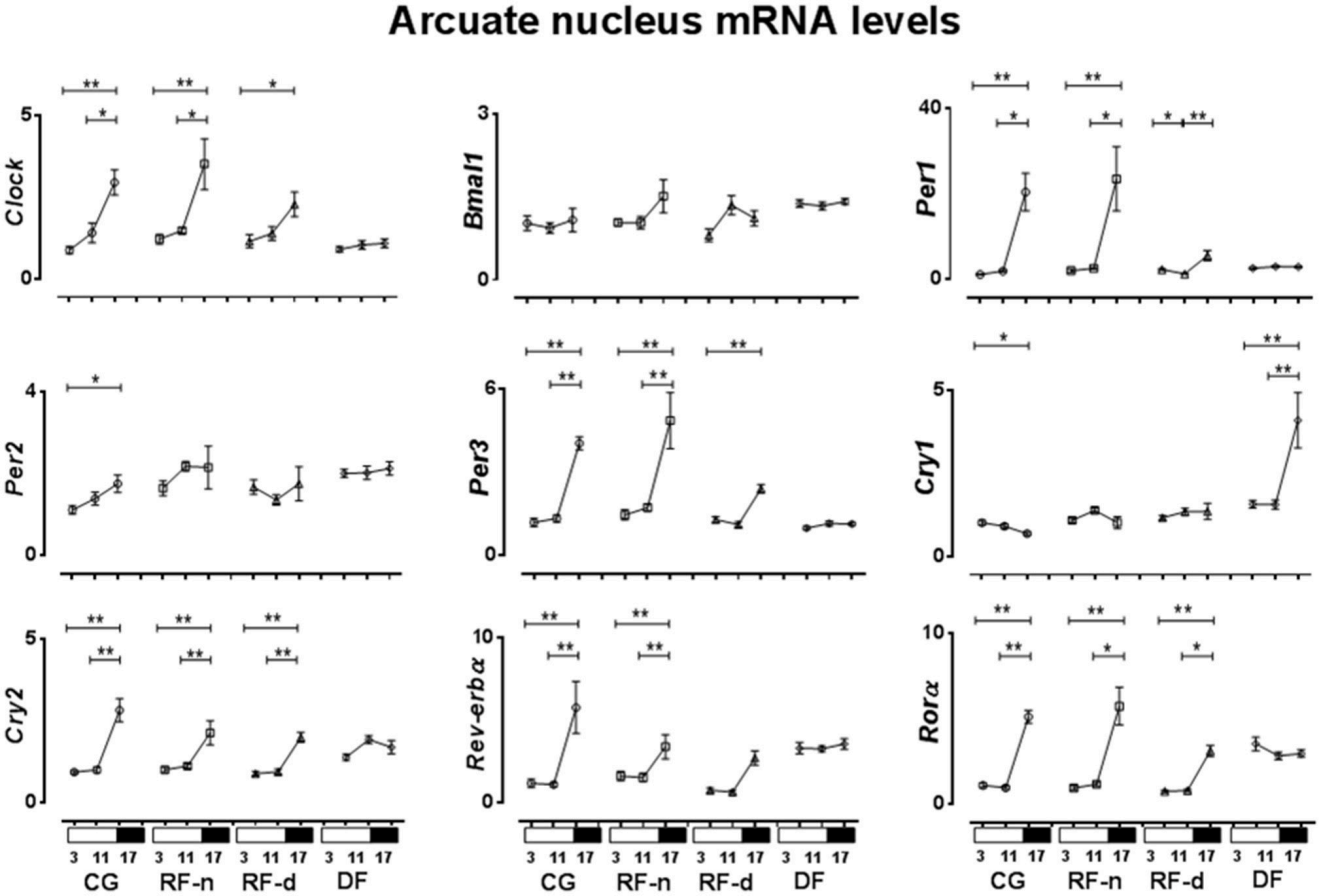
**Relative expression of clock genes in Arcuate nucleus (ARC) in the control group, with food ***ad libitum***; restricted night-fed group (RF-night), with access to food restricted to ZT12–ZT14 (1800–2000 h); restricted day-fed group (RF-day), with access to food restricted to ZT3–ZT5 (0900–1100 h); and day-fed group (DF), with access to food restricted to ZT0–ZT12 (0600–1800 h) at ZT3, ZT11, and ZT17, corresponding to 0900, 1700, and 2300 h; respectively**. *N* = 6–10 rats per time point per group. ZT, Zeitgeber Time. Significant difference among ZTs within the same feeding condition: ^*^P < 0.05, ^**^P < 0.01.

The expression of clock genes in the ARC of CG, RF-n, RF-d, and DF groups at ZT3, ZT11, and ZT17 are presented in Figure 5. No difference in *Bmal1* expression was observed among ZT3, ZT11, and ZT17 in any group. In the CG, *Cry1* expression was higher (*P* = 0.02) in the morning (ZT3), while *Clock, Per1, Per2, Per3, Cry2, Rev-erb*α, and *Ror*α (*P* < 0.01) showed higher expression at night (ZT17). Compared with CG, RF-n and RF-d groups showed similar pattern of expression of *Clock* (*P* < 0.01 and *P* = 0.04), *Per3* (*P* < 0.01 and *P* < 0.01), *Cry2* (*P* < 0.01 and *P* < 0.01), and *Ror*α (*P* < 0.01 and *P* < 0.01), while the pattern of expression of *Per2* and *Cry1* was lost. RF-n also had higher *Per1* expression at ZT17 (*P* < 0.01), while RF-d exhibited higher *Per1* expression at morning (ZT3) and at night (ZT17) (*P* < 0.01). RF-d group maintained the pattern of *Rev-erb*α expression observed in CG group, while RF-n lost this pattern. DF group presented higher expression of *Cry1* at ZT17 than at ZT3 and ZT11 (*P* < 0.01).

### The feeding restriction schedules and the modulation of temporal relationship of clock gene expression with corticosterone secretion

In the SCN of control animals, the higher *Per2* expression was concomitant with the higher corticosterone secretion while no relationship was observed regarding *Clock, Bmal1, Per1, Per3, Cry1, Cry2, Rev-erb*α, and *Ror*α genes. In the RF-n group, besides *Per2*, the expression of *Clock, Per1*, and *Per3* genes also exhibited higher expression coinciding with higher corticosterone secretion. The same pattern was observed for *Per1* and *Per3* in RF-d group, in which there was also a delay of the higher expression of *Clock, Bmal1, Per2, Cry1, Cry2*, and *Ror*α compared with the higher corticosterone secretion. On the other hand, in DF group the most interesting finding was the anticipatory higher expression of *Clock* and *Bmal1* regarding the corticosterone secretion.

In the PVN of control animals, the higher expression of *Per1, Per2, Per3, Cry2*, and *Ror*α were concomitant with the higher corticosterone secretion, while no relationship was observed regarding *Clock, Bmal1, Cry1*, and *Rev-erb*α genes. In the RF-n, the higher expression of *Per2, Per3*, and *Ror*α was delayed concerning the corticosterone secretion, while there was no relationship regarding the expression of *Clock, Bmal1, Per1, Cry1, Cry2*, and *Rev-erb*α genes. On the other hand, in the RF-d and DF groups, the majority of the studied genes lost their relationship with corticosterone secretion compared with the pattern observed in control and RF-n groups. Of note, in DF group, the higher expression of *Ror*α and *Rev-erb*α was anticipatory concerning corticosterone secretion.

In the ARC of control animals, while the higher expression of *Clock, Per1, Per2, Per3, Cry2, Rev-erb*α, and *Ror*α was coincident, the *Cry1* higher expression was anticipatory in relation to corticosterone secretion. No relationship was observed regarding *Bmal1* gene. On the other hand, in the RF-n and RF-d groups, there was a delay of the higher expression of *Clock, Per1, Per3, Cry2, Rev-erb*α, and *Ror*α compared with corticosterone secretion. No relationship was observed concerning *Bmal1, Cry1*, and *Per2* in RF-n group and *Rev-erb*α in RF-d group, respectively. Of note, in DF group, most of the genes lost the pattern of expression, also missing any relationship with corticosterone secretion.

## Discussion

In the present study, rats submitted to three different schedules of food restriction changed the diurnal pattern of *Per1, Per2*, and *Per3* expression in the SCN and in the extra-SCN oscillators—PVN and ARC nuclei—suggesting that non-photic cues can modulate the expression of clock genes, but not in a uniform way, throughout the hypothalamic nuclei. Furthermore, the corticosterone anticipatory peak was verified irrespective to the food timing access, except in the DF group. Altogether our data confirm that restricted feeding schedules can be an entrainment cue for glucocorticoid circadian variation.

The initial body weight of rats was similar in all groups, but at the end of the experiment, RF-d and RF-n groups showed lower weight as well as lower daily food intake than control and DF groups, similarly to studies using food restriction but with different duration of food access (Honma et al., 1983; Stephan and Becker, 1989; Stephan, 2002). In our study, the time of feeding in RF-d and RF-n groups was restricted by 2 h of food access leading to about 50% calorie intake restriction when compared with controls, suggesting that body weight could be determined primarily by the amount of energy intake and not by the time of feeding. Indeed, control and DF groups showed similar body weight. Arble et al. (2009) showed that mice fed during the 12-h light phase gained even more weight than mice fed on an equivalent amount of calories during the 12-h dark phase (Arble et al., 2009). Of note, in that study, differently from our study, mice were fed with high-fat diet during 6 weeks. Altogether, these data point out that a longer extent of daytime feeding would be necessary to achieve higher body weight.

Change in the daily eating pattern is considered a powerful Zeitgeber for the diurnal rhythm of glucocorticoids (Krieger, 1974; Leal and Moreira, 1996; Girotti et al., 2009). Higher corticosterone concentrations were observed in the control and RF-n groups at ZT11 than at ZT3, coincident with the onset of the nocturnal activity period of rats (Le Minh et al., 2001). On the other hand, RF-d group, which was allowed to eat only during 2 h in the morning, showed higher concentrations of corticosterone at ZT3 than at ZT11, inverting the rat corticosterone circadian pattern, as previously described (Krieger, 1974; Moreira and Krieger, 1982; Leal et al., 1995; Leal and Moreira, 1996). Of note, in the RF-n group, in which food access was restricted but occurred without dissociation with the pattern of rat nocturnal activity, we observed an entrainment of corticosterone peak coincident with food availability. Furthermore, the ZT3 and ZT11 time points allowed us to observe the well known anticipatory corticosterone peak before food availability during the light phase of the light/dark cycle (Oliveira et al., 1993; Le Minh et al., 2001). This finding was not observed in the DF group, which presented higher corticosterone levels at ZT11 as control and RF-n groups. Our data are in agreement with previous reports showing that anticipatory corticosterone peak did not happen when food availability occurs during longer intervals of the day (Honma et al., 1983; Belda et al., 2005). Probably, in this situation the photic cue continues as the main Zeitgeber.

In the SCN, all groups of restricted feeding schedules exhibited different patterns of the expression of *Clock* and *Per1* genes when compared to the control group, while the *Per3* gene expression was only modified in the RF-d and RF-n groups, characterized by an intense food deprivation (food availability restricted by 2 h). The expression of *Bmal1, Cry1*, and *Cry2* was only modified in the RF-d and DF groups, which food access occurred in dissociation with the pattern of rat nocturnal activity, presenting higher expression in the morning, and early evening. In contrast, a higher expression of *Per2* at ZT11 than at ZT17 was observed in all groups, thus the expression of this gene was unchanged by variable food schedule. Our data demonstrated, in agreement with Caldelas et al. (2005) and Minana-Solis et al. (2009) but in contrast to others (Damiola et al., 2000; Hara et al., 2001; Wakamatsu et al., 2001; Verwey et al., 2007) that SCN is also affected by food entrainment. This controversial data can be explained mainly because of the period in which rats were food entrained in the different protocols, as well as by the duration of daily food access, which varied from 2 h, similar to our study, as long as 12 h as established by Damiola et al. (2000). Thus, time of the day as well as the duration of daily food access may modulate the SCN response, as previously suggested by Minana-Solis et al. (2009). Therefore, feeding cycle can be considered the dominant Zeitgeber for the clock gene expression not only in the peripheral but also in the central pacemakers.

We were not able to detect daily variations in the expression of clock genes in the SCN of the control group, except for *Per2*. Our protocol explored three time points only, which could be a possible limitation to properly study variations in circadian rhythm but it is still informative to understand the role of restricted feeding on the expression of clock genes in hypothalamic nuclei. However, in the PVN and ARC, higher expressions of *Per2, Per3*, and *Cry2* were observed synchronically at ZT17 in the dark period, whereas the other clock genes phased differently. In addition, different food schedules changed the expression of some clock genes in the SCN, PVN, and ARC, compared with CG. Thus, our data demonstrated that under *ad libitum* food access or under food restriction there are different phases in the clock gene expression between the SCN and extra-SCN nuclei as PVN and ARC hypothalamic nuclei.

Data on ARC and in other energy balance and feeding behavior related areas have shown that dietary restriction induces or increases the amplitude of the daily rhythms of *Per1* and *Per2* genes and their proteins (Mieda et al., 2006; Verwey et al., 2007; Angeles-Castellanos et al., 2008; Guilding et al., 2009). In addition, c-*fos, Per1, Per2*, and *Bmal1* rhythms were in antiphase with the respective SCN genes in the PVN in rats under food restriction, suggesting that extra-SCN neuronal clocks can be, but not always, expressed in antiphase from the SCN (Damiola et al., 2000). Another study also showed that the daily food restriction did not lead to a uniform timing effect in clock gene expression in the different hypothalamic nuclei (Minana-Solis et al., 2009). Our data expand these findings since we studied a larger number of clock genes and three different food restriction schedules. Indeed, RF-n, RF-d, and DF groups also showed differential effects on the expression of clock genes, inducing phase alteration in some cases or keeping them unaffected in others. Of note, daily restricted feeding can produce food-entrainable oscillations in the cerebral cortex, hippocampus, striatum, pyriform cortex, and PVN even in SCN-lesioned animals (Wakamatsu et al., 2001). Thus, clock genes in extra-SCN oscillators do not respond to feeding schedules in a uniform manner, suggesting a tissue-specific regulation (Feillet et al., 2008; Minana-Solis et al., 2009).

Our data regarding the modulation of feeding restriction schedules on temporal relationship between clock gene expression and corticosterone secretion show that the most important changes were observed in RF-d and DF groups, in which conditions the eating pattern was not coincident with the onset of the nocturnal activity period, suggesting that a disruption in feeding cues may cause dissociation between gene expression and corticosterone secretion. This misalignment can explain night eating syndrome observed in humans, which is characterized by increased food consumption late in the day, in the evening, and in the night and has been linked to obesity and increased risk of metabolic syndrome (Nader et al., 2010).

In conclusion, our data show that feeding restriction schedules induce changes in the body weight, in the corticosterone circadian variation, and in the expression of the clock genes in hypothalamic nuclei, suggesting the ability of animals to predict, anticipate, and respond to food availability. The pattern of clock gene expression in response to RF-n, RF-d, and DF schedules was not affected uniformly, suggesting that the master oscillator in SCN as well as PVN and ARC, areas involved in energy homeostasis and homeostatic aspect of feeding, are regulated in a tissue-specific manner in response to feeding restriction, a non-photic cue, in order to entrain clock gene system.

## Author contributions

LD: Conception and design of research, performed experiments, analyzed data, interpreted results of experiments, prepared figures, drafted manuscript, edited, and revised manuscript, approved final version of manuscript. SR: Performed experiments, interpreted results of experiments, prepared figures, revised manuscript and approved final version of manuscript. AB: Performed experiments, analyzed data and interpreted results of experiments. FC: Performed experiments, analyzed data and interpreted results of experiments. CM: Performed experiments, analyzed data and interpreted results of experiments, revised manuscript and approved final version of manuscript. EU: Conception and design of research, performed experiments and interpreted results of experiments. JA: Conception and design of research, revised manuscript, approved final version of manuscript. LE: Conception and design of research, revised manuscript, approved final version of manuscript. PE: Conception and design of research, revised manuscript, approved final version of manuscript. AM: Conception and design of research, analyzed data, interpreted results of experiments, edited and revised manuscript, approved final version of manuscript. MC: Conception and design of research, analyzed data, interpreted results of experiments, prepared figures, drafted manuscript, edited and revised manuscript, approved final version of manuscript. I certify that all authors had a substantial contribution to the manuscript.

## Funding

National Counsel of Technological and Scientific Development—CNPq, Coordination for the Improvement of Higher Level Personnel—CAPES, Sao Paulo Research Foundation—FAPESP (Process numbers: 2007/58365-3, 2013/09799-1).

### Conflict of interest statement

The authors declare that the research was conducted in the absence of any commercial or financial relationships that could be construed as a potential conflict of interest.
